# Supplementary material for: The Effect of Alkali Iodide Salts in the Inclusion Process of Phenolphthalein in β-Cyclodextrin: A Spectroscopic and Theoretical Study
Source: Molecules. 2023 Jan 23;28(3):1147. doi: 10.3390/molecules28031147 (PMC9920586; doi:10.3390/molecules28031147)
Supplement: Supplementary file 1 [file molecules-28-01147-s001.zip › molecules-2127231-supplementary.docx]

**Table S1.** Molar concentrations of the prepared solutions.

|  | β-CD (×10^-5^ M) | PP (×10^-5^ M) | LiI (M) | NaI (M) | KI (M) | CsI (M) |
| --- | --- | --- | --- | --- | --- | --- |
| **Sample 1** | 0 | 5.0 | **-** | **-** | **-** | **-** |
| **Sample 2** | 3.3 | 5.0 | **-** | **-** | **-** | **-** |
| **Sample 3** | 6.6 | 5.0 | **-** | **-** | **-** | **-** |
| **Sample 4** | 9.9 | 5.0 | **-** | **-** | **-** | **-** |
| **Sample 5** | 13.2 | 5.0 | **-** | **-** | **-** | **-** |
| **Sample 6** | 16.5 | 5.0 | **-** | **-** | **-** | **-** |
| **Sample 7** | 19.8 | 5.0 | **-** | **-** | **-** | **-** |
| **Sample 8** | 0 | 5.0 | 0.1 | **-** | **-** | **-** |
| **Sample 9** | 3.3 | 5.0 | 0.1 | **-** | **-** | **-** |
| **Sample 10** | 6.6 | 5.0 | 0.1 | **-** | **-** | **-** |
| **Sample 11** | 9.9 | 5.0 | 0.1 | **-** | **-** | **-** |
| **Sample 12** | 13.2 | 5.0 | 0.1 | **-** | **-** | **-** |
| **Sample 13** | 0 | 5.0 | **-** | 0.1 | - | - |
| **Sample 14** | 3.3 | 5.0 | **-** | 0.1 | - | - |
| **Sample 15** | 6.6 | 5.0 | **-** | 0.1 | - | - |
| **Sample 16** | 9.9 | 5.0 | **-** | 0.1 | - | - |
| **Sample 17** | 13.2 | 5.0 | **-** | 0.1 | - | - |
| **Sample 18** | 0 | 5.0 | **-** | **-** | 0.01 | **-** |
| **Sample 19** | 3.3 | 5.0 | **-** | **-** | 0.01 | **-** |
| **Sample 20** | 6.6 | 5.0 | **-** | **-** | 0.01 | **-** |
| **Sample 21** | 9.9 | 5.0 | **-** | **-** | 0.01 | **-** |
| **Sample 22** | 13.2 | 5.0 | **-** | **-** | 0.01 | **-** |
| **Sample 23** | 0 | 5.0 | **-** | **-** | 0.05 | **-** |
| **Sample 24** | 3.3 | 5.0 | **-** | **-** | 0.05 | **-** |
| **Sample 25** | 6.6 | 5.0 | **-** | **-** | 0.05 | **-** |
| **Sample 26** | 9.9 | 5.0 | **-** | **-** | 0.05 | **-** |
| **Sample 27** | 13.2 | 5.0 | **-** | **-** | 0.05 | **-** |
| **Sample 28** | 0 | 5.0 | **-** | **-** | 0.1 | **-** |
| **Sample 29** | 3.3 | 5.0 | **-** | **-** | 0.1 | **-** |
| **Sample 30** | 6.6 | 5.0 | **-** | **-** | 0.1 | - |
| **Sample 31** | 9.9 | 5.0 | **-** | **-** | 0.1 | - |
| **Sample 32** | 13.2 | 5.0 | **-** | **-** | 0.1 | - |
| **Sample 33** | 0 | 5.0 | **-** | **-** | 0.2 | - |
| **Sample 34** | 3.3 | 5.0 | **-** | **-** | 0.2 | - |
| **Sample 35** | 6.6 | 5.0 | **-** | **-** | 0.2 | **-** |
| **Sample 36** | 9.9 | 5.0 | **-** | **-** | 0.2 | **-** |
| **Sample 37** | 13.2 | 5.0 | **-** | **-** | 0.2 | **-** |
| **Sample 38** | 0 | 5.0 | **-** | **-** | **-** | 0.2 |
| **Sample 39** | 3.3 | 5.0 | **-** | **-** | **-** | 0.2 |
| **Sample 40** | 6.6 | 5.0 | **-** | **-** | **-** | 0.2 |
| **Sample 41** | 9.9 | 5.0 | - | **-** | **-** | 0.2 |
| **Sample 42** | 13.2 | 5.0 | - | - | - | 0.2 |
